# WO_3_/BiVO_4_ Photoanodes: Facets Matching at the Heterojunction and BiVO_4_ Layer Thickness Effects

**DOI:** 10.1021/acsaem.1c01623

**Published:** 2021-08-12

**Authors:** Ivan Grigioni, Giovanni Di Liberto, Maria Vittoria Dozzi, Sergio Tosoni, Gianfranco Pacchioni, Elena Selli

**Affiliations:** † Dipartimento di Chimica, Università degli Studi di Milano, Via Golgi 19, 20133 Milano, Italy; ‡ Dipartimento di Scienza dei Materiali, Università di Milano-Bicocca, Via Cozzi 55, 20125 Milano, Italy

**Keywords:** photoelectrochemistry, semiconductor interface, band alignment, density functional theory, WO_3_, BiVO_4_

## Abstract

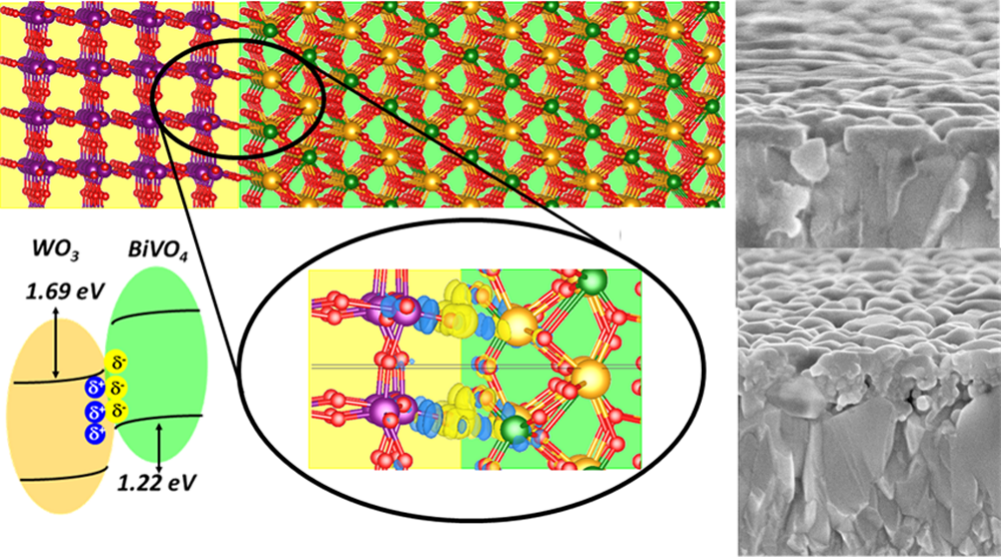

Photoelectrochemical solar energy conversion offers a way to directly store light into energy-rich chemicals. Photoanodes based on the WO_3_/BiVO_4_ heterojunction are most effective mainly thanks to the efficient separation of photogenerated charges. The WO_3_/BiVO_4_ interfacial space region in the heterojunction is investigated here with the increasing thickness of the BiVO_4_ layer over a WO_3_ scaffold. On the basis of X-ray diffraction analysis results, density functional theory simulations show a BiVO_4_ growth over the WO_3_ layer along the BiVO_4_ {010} face, driven by the formation of a stable interface with new covalent bonds, with a favorable band alignment and band bending between the two oxides. This crystal facet phase matching allows a smooth transition between the electronic states of the two oxides and may be a key factor ensuring the high efficiency attained with this heterojunction. The photoelectrochemical activity of the WO_3_/BiVO_4_ photoanodes depends on both the irradiation wavelength and the thickness of the visible-light-absorbing BiVO_4_ layer, a 75 nm thick BiVO_4_ layer on WO_3_ being best performing.

## 1 Introduction

Photoelectrochemical (PEC) water splitting is a promising route to directly generate hydrogen from water and solar light, and hydrogen is regarded as a clean energy vector, enabling the storage of the intermittent renewable solar energy. A solar-to-hydrogen (STH) conversion efficiency of 10% is considered as a threshold for industrialization.^1^ Photoanodes for the oxygen evolution reaction (OER) based on metal oxides offer good stability and light harvesting over the whole visible spectrum.^2−5^

In this scenario, BiVO_4_ rapidly emerged as one of the most promising visible-light-active photoanode materials. State-of-the-art BiVO_4_ electrodes display remarkable stability above 1000 h^6,7^ and a STH conversion efficiency of 5%.^8^ The deposition of OER catalysts allows to decrease the overpotential needed to speed up the slow O_2_ evolution kinetics, thus boosting the electrode performance.^9−11^ However, a full utilization of the photons absorbed by BiVO_4_ is hampered by the recombination of photogenerated charges and several strategies, such as doping,^12−15^ gradient doping,^8^ reduction by H_2_ treatment,^16^ and heterojunction engineering,^17^ have been developed to mitigate such a detrimental energy loss path.

Photoanodes based on the WO_3_/BiVO_4_ heterojunction showed a record STH conversion efficiency^18,19^ owing to the beneficial combination of the excellent visible-light-harvesting properties of BiVO_4_ and the superior electron conductivity of WO_3_.^20−22^ Indeed, resistance to charge transfer largely diminishes when BiVO_4_ is combined with WO_3_, as demonstrated by electrochemical impedance spectroscopy measurements.^17^ Furthermore, the in-built electric field generated by the favorable band energy offset between the two oxides^23^ drives efficient separation of photogenerated electron–hole pairs since holes remain in the BiVO_4_ valence band (VB),^24,25^ while photopromoted electrons flow into the lower-lying WO_3_ conduction band (CB), where they rapidly diffuse to the external circuit, exploiting the better charge mobility within this material.^26^ This enhanced spatial charge separation occurring in WO_3_/BiVO_4_ heterojunctions leads to relatively long-living charge carriers^27,28^ and to efficient light harvesting.^18,19^

Although the complex charge-carrier dynamics in this system has been studied by different groups in very recent years,^24,28−32^ the crystal growth of BiVO_4_ at the interface with WO_3_ during the heterojunction formation, which may have a key role in determining the outstanding charge separation of this architecture, is so far little explored. A recent study reveals, however, that the crystallographic orientation of the underlying WO_3_ layer may affect the PEC performance of WO_3_/BiVO_4_ heterojunctions.^33^

In the present work, we investigate the effect that the thickness of the visible-light-absorbing BiVO_4_ layer has on the PEC performance of the photoanodes, further complementing the experimental results with first-principles calculations. In particular, two series of six photoanodes were prepared through a wet chemistry synthetic method. The first series consists of BiVO_4_ layers of variable thicknesses directly deposited on a fluorine-doped tin oxide (FTO) conductive glass, while the second one was obtained by the deposition of the same BiVO_4_ layers on a WO_3_ layer supported on FTO. In both series, only the thickness of the BiVO_4_ layer was varied while maintaining that of WO_3_ fixed. The morphological and crystallographic features of the two photoanode series were investigated in relation to their PEC performance. Density functional theory (DFT) simulations allowed us to identify specific facet interaction between the two oxides, which may guide and control the progressive crystalline growth at the heterojunction and create a stable interface with a type-II band alignment. In addition, the formation of an interface polarization in the contact region between the two oxides leads to a band bending that favors charge-carrier separation.

## 2 Experimental Section

### 2.1 Materials

The following chemicals were employed: tungsten(VI) ethoxide 99.8% (5% w/v in ethanol), ammonium vanadium oxide, bismuth(III) nitrate pentahydrate ACS 98%, benzyl alcohol ACS 99% (Alpha Aesar), ethyl cellulose (MP Biomedics), citric acid 99% and zirconium oxynitrate hydrate 99% (Aldrich), and anhydrous sodium sulfate (Fisher Scientific).

### 2.2 Photoelectrode Preparation

The WO_3_ layer was prepared as follows: 1.0 mL of tungsten ethoxide, 5 wt % in ethanol, was added to 42 mg of citric acid acting as a stabilizer. Once citric acid was completely dissolved, benzyl alcohol (0.3 mL) and ethyl cellulose (40 mg) were added to the solution and stirred overnight to allow the complete dissolution of ethyl cellulose. The so-obtained paste is stable for several weeks. FTO glass (Pilkington Glass, TEC-7, thickness 2 mm) was coated with the paste by spin-coating at 4000 rpm for 30 s. The final spinning rate was reached with a three acceleration step program: 200 rpm s^–1^ up to 1000 rpm, then 500 rpm s^–1^ up to 2000 rpm, and finally 2000 rpm s^–1^ up to 4000 rpm. Prior to deposition, the FTO glass was cleaned by a 30 min long sonication in an aqueous soap solution, then in ethanol, and finally in water. After coating, the film was dried at 80 °C for 1 h and then annealed at 500 °C for 8 h.

Bismuth vanadate films were prepared starting from a liquid solution similar to that reported elsewhere.^34^ In a typical synthesis, 0.002 mol of Bi(NO_3_)_3_ and NH_4_VO_3_ were added to 6 mL of HNO_3_ 23.3% containing 0.004 mol citric acid. The mixture was stirred overnight to allow dissolution of the precursor. The BiVO_4_-based photoanodes were prepared on clean FTO by spinning the solution at 8000 rpm for 30 s with an acceleration rate of 6000 rpm s^–1^. The film was then dried for 1 h at 80 °C and calcined for 1 h at 500 °C. The thickness of the BiVO_4_ film was controlled by depositing successive coating layers by repeating the spin-coating procedure and the thermal treatment up to eight times. Once the desired optical density of the film was obtained, the electrode was annealed at 500 °C for 8 h.

The WO_3_/BiVO_4_ combined photoanodes with different thicknesses were prepared by coating the WO_3_ electrodes (prepared as described above) with the BiVO_4_ precursor solution. Then, the composite film was dried at 80 °C for 1 h and annealed at 500 °C for 1 h. The amount of BiVO_4_ in the WO_3_/BiVO_4_ electrodes was controlled in the same way as for the BiVO_4_ photoanode series. The films were finally annealed at 500 °C for 8 h.

Using the here described successive BiVO_4_ layer deposition technique, we effectively tailored the thickness of the BiVO_4_ compart, obtaining a comparable BiVO_4_ thickness in the BiVO_4_ and WO_3_/BiVO_4_ electrodes prepared by applying the same number of coated layers (the same optical densities were obtained in the wavelength region where only BiVO_4_ absorbs light). The two series of photoanodes were labeled as BiVO_4_-*X* and WO_3_/BiVO_4_-*X*, with *X* indicating the BiVO_4_ layer thickness expressed in nanometers (*X* = 15, 30, 50, 75, 115, and 160). Each actual BiVO_4_ thickness value was slightly approximated to simplify the direct comparison between the films of the two series containing the same number of BiVO_4_-coated layers. Please refer to Table S1 of the Supporting Information for the exact film thickness values.

A zirconia film deposited on FTO, prepared starting from ZrO(NO_3_)_2_ with a procedure similar to that employed for the preparation of BiVO_4_ films (i.e., same precursors concentration, deposition rate, and annealing temperature) was employed, instead of FTO, to record the baseline when recording the absorption spectra of the FTO/BiVO_4_ photoanode series in the transmittance mode. In fact, when FTO was employed to record the baseline, negative absorbance values were obtained for the thinner FTO/BiVO_4_ electrodes due to considerable light scattering by pristine FTO, exhibiting an average absorbance of ca. 0.2 above 400 nm. On the other hand, upon covering FTO with the wide band gap thin ZrO_2_ layer, this scattering phenomenon significantly decreased.

### 2.3 Optical, Morphological, and Structural Characterization

Images showing the morphology and the cross-sectional view of the electrodes were obtained using a FEI Magellan-400 field emission scanning electron microscope. UV–visible absorption spectra were recorded using a Jasco V650 spectrophotometer. The crystalline phase of the materials was determined through X-ray diffraction (XRD) analysis using a Philips PW1820 instrument with Cu Kα radiation at 40 mA and 40 kV. The diffractograms were base-corrected employing Origin software.

### 2.4 PEC Characterization

PEC measurements were carried out using a three-electrode cell with an Ag/AgCl (3.0 M NaCl) reference electrode, a platinum gauze as a counter electrode, and a Princeton Applied Research 2263 (PARstat) potentiostat. The light source was a 300 W Xe lamp with an AM 1.5 G illumination (1 sun). A 0.5 M Na_2_SO_4_ aqueous solution was used in electrochemical measurements. The potential versus Ag/AgCl was converted into the RHE scale using the following equation: *E*_RHE_ = *E*_AgCl_ + 0.059 pH + *E*_AgCl_°, with *E*_AgCl_° (3.0 M NaCl) = 0.210 V at 25 °C. Linear sweep voltammetry (LSV) scans were recorded at 10 mV s^–1^ under backside irradiation (irradiation on the FTO side), starting from the open-circuit potential after 5 min of irradiation, up to 1.8 V versus RHE.

Incident photon-to-current efficiency (IPCE) measurements were carried out with a setup similar to that used for PEC experiments, with a Bausch and Lomb grating monochromator placed between the Xe lamp and the sample. A 1.23 V bias versus NHE was applied and the current was measured with a 10 nm step, within the 350–600 nm wavelength range. The incident light power was measured at each wavelength using a calibrated photodiode connected to a Keithley 617 electrometer. The IPCE was calculated at each wavelength λ (nm) using the following equation
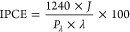
where *J* is the photocurrent density (mA cm^–2^) and *P*_λ_ (mW cm^–2^) is the power of the monochromatic light at the specific wavelength λ.

### 2.5 Computational Methods

Calculations were performed at the DFT level by means of the CRYSTAL code.^35^ For the exchange and correlation potential, we recurred to the HSE06 range-separated hybrid functional (25% fraction of Fock exchange),^36^ a suitable choice for the treatment of solids.^37,38^ The all-electron Gaussian-type basis sets 8-411(d1) and 86-411(d31) were adopted for O and V.^39^ For the heavy tungsten and bismuth atoms, we adopted the effective core pseudopotential (ECP) by Hay and Wadt, with a modified double-zeta basis set for valence electrons.^40,41^ The valence electrons treated explicitly are 5p, 6s, and 5d for W and 6s and 6p for Bi. These basis sets were already used and benchmarked for the study of these materials.^42−44^ In particular, the adopted basis set of WO_3_ has been used and discussed in extended studies conducted on both bulk and surfaces in the past.^42,43^ In the case of BiVO_4_, we benchmarked the performances of the adopted basis sets against available flexible TZVP basis functions.^45^ The impact on the electronic band gaps of bulk phases induces differences as high as 0.1 eV and only 0.1% on the lattice parameters. Bulk, surface, and interface models were fully optimized. Full details on cutoff limits, convergence criteria and thresholds, and reciprocal space sampling are reported in the Supporting Information.

The choice of the functional used and the mismatch induced in forming a heterojunction are among the approximations that affect the absolute values of the computed properties. About the first point, it should be mentioned that the BiVO_4_ band gap computed with hybrid functionals is overestimated.^38,46^ This opens the question of the functional to be used for the WO_3_/BiVO_4_ junction. In a seminal study on the band alignment in ZnO/TiO_2_, Conesa used the average value between the optimal exchange fractions derived for the isolated ZnO and TiO_2_ components.^47^ This strategy performs well in general. However, the optimal exchange fractions to reproduce the experimental band gap of BiVO_4_ and WO_3_ are ∼0% and ∼0.22, respectively, and the average value for the heterostructure should be ∼0.1. This could result in very different band alignments for the real BiVO_4_/WO_3_ interface and for that derived for the independent species. Another strategy consists in using the same DFT functional for the separated components and the heterojunction. This procedure leads to an error that is transferrable from independent units’ calculations to heterojunction ones and therefore can be better rationalized. We adopted this second strategy.

The problem of mismatch has been studied in detail in this and other studies, with the observation that a strain of the entity present in our systems can result in about 0.2 eV error in the position of the band edges. In the present case, the adopted simulation cell implies lattice mismatches of 1.2 and 2.1% on *a* and *b* lattice vectors, respectively, leading to deviations of up to 0.1 eV in the position of the band edges. This is not going to affect the conclusions of this work. It is worth recalling that the simulation of interface models of this type with DFT methods implies the approximation that the structure of the interface is sharp to obey to the periodic boundary conditions, that is, dislocation and amorphization effects are not included in the simulation.

## 3 Results and Discussion

### 3.1 Morphological, Optical, and Structural Characterization

**Figure 1:**
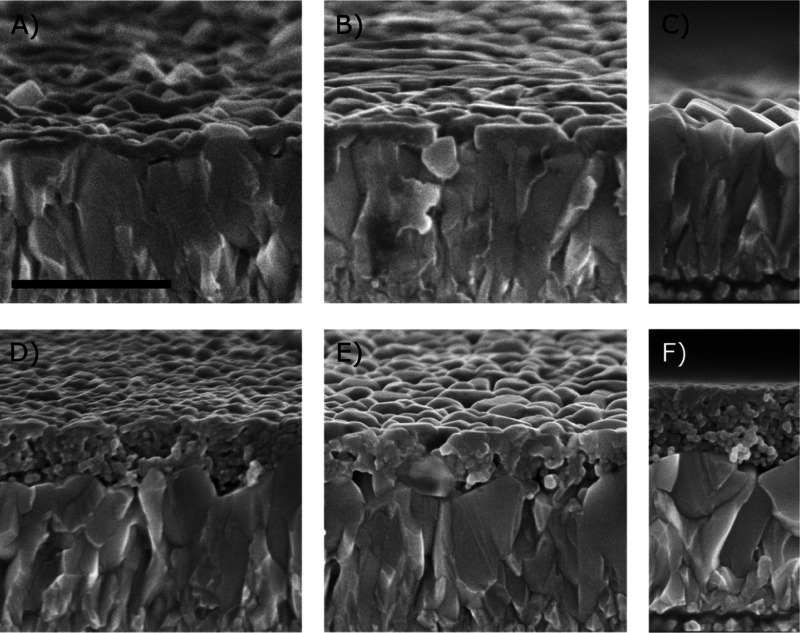
FESEM side-view images of the photoanodes obtained by the successive deposition of (A,D) two and (B,E) four BiVO_4_ layers, (A,B) directly on the FTO conductive glass (films BiVO_4_-30 and BiVO_4_-75, respectively), and (D,E) on a 200 nm thick WO_3_ layer (films WO_3_/BiVO_4_-30 and WO_3_/BiVO_4_-75, respectively); representative cross sections of (C) the FTO and (F) the WO_3_ on the FTO film. The scale bar is 500 nm.

Side-view field emission scanning electron microscopy (FESEM) images of the BiVO_4_ photoanodes obtained upon the deposition of two and four BiVO_4_-coated layers and that of the FTO glass used as support, together with those of the corresponding WO_3_/BiVO_4_ photoanodes and of the WO_3_ photoanode, are reported in Figure 1.

Figure 1A shows that the asperities of the underlying FTO conductive glass still emerge from the thinnest BiVO_4_ film in the BiVO_4_-30 photoanode, while they are completely covered upon further deposition of two BiVO_4_ layers in BiVO_4_-75 (Figure 1B). On the other hand, the deposition of a ca. 200 nm thick WO_3_ layer on FTO led to a flat surface (Figure 1F), and the deposition of only two BiVO_4_ layers ensured a homogeneous coverage of the FTO/WO_3_ underlying film in the WO_3_/BiVO_4_-30 photoanode (Figure 1D), while larger crystallites were obtained upon the deposition of four BiVO_4_ layers onto WO_3_ (see the image referring to WO_3_/BiVO_4_-75 in Figure 1E). In both photoanode series, successive BiVO_4_ depositions led to an increase in size of the BiVO_4_ grains, forming a mesoporous architecture.

**Figure 2:**
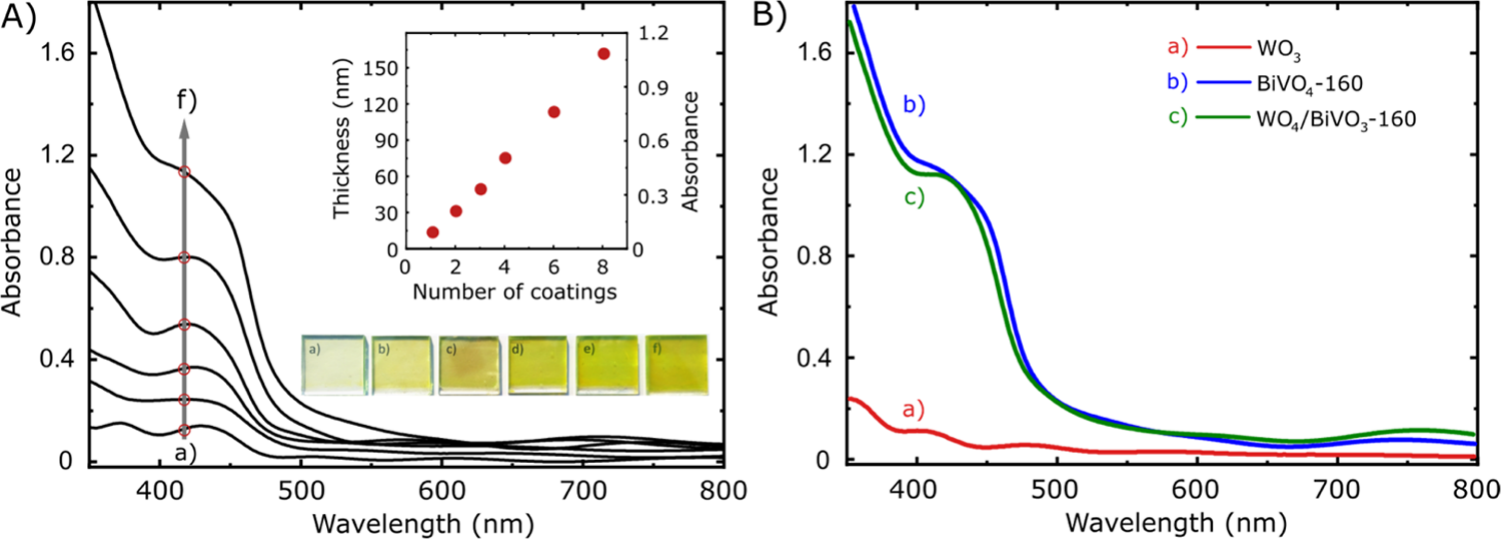
(A) Absorption spectra of the BiVO_4_ series obtained by the successive deposition of (from a to f) one, two, three, four, six, and eight BiVO_4_-coated layers; insets: thickness and absorbance increase (left and right ordinate axis, respectively) vs the number of BiVO_4_-coated layers and corresponding pictures of the photoanode series. (B) Absorption spectra of the oxide films deposited on FTO: (a) WO_3_; (b) BiVO_4_-160; and (c) WO_3_/BiVO_4_-160.

The absorption spectra of the BiVO_4_ photoanode series are reported in Figure 2A, together with their picture. The absorbance linearly increases with the number of BiVO_4_-coated layers, as shown in the inset of Figure 2A. From the thickness of the two-, four-, and six-layered BiVO_4_ films, evaluated from the cross-sectional images reported in Figure S1 of the Supporting Information, and the absorption spectra of these films, the absorption coefficient of BiVO_4_ at 420 nm, α_420_ = 6.7 10^4^ cm^–1^, was calculated, as detailed in the Supporting Information. This value was employed to estimate the thickness of the BiVO_4_ layer in WO_3_/BiVO_4_ photoanodes.

The absorption spectra of the WO_3_, BiVO_4_-160, and WO_3_/BiVO_4_-160 films are shown in Figure 2B. Notably, the successive spin-coating and annealing procedure ensured reproducible results in terms of absorbance and film thickness increase on both FTO and FTO/WO_3_ substrates also after eight layer depositions, as indicated by the almost superimposed absorption profiles of the BiVO_4_-160 and WO_3_/BiVO_4_-160 systems. Residual apparent light absorption above 520 nm is mainly due to lattice fringes and light scattering.

**Figure 3:**
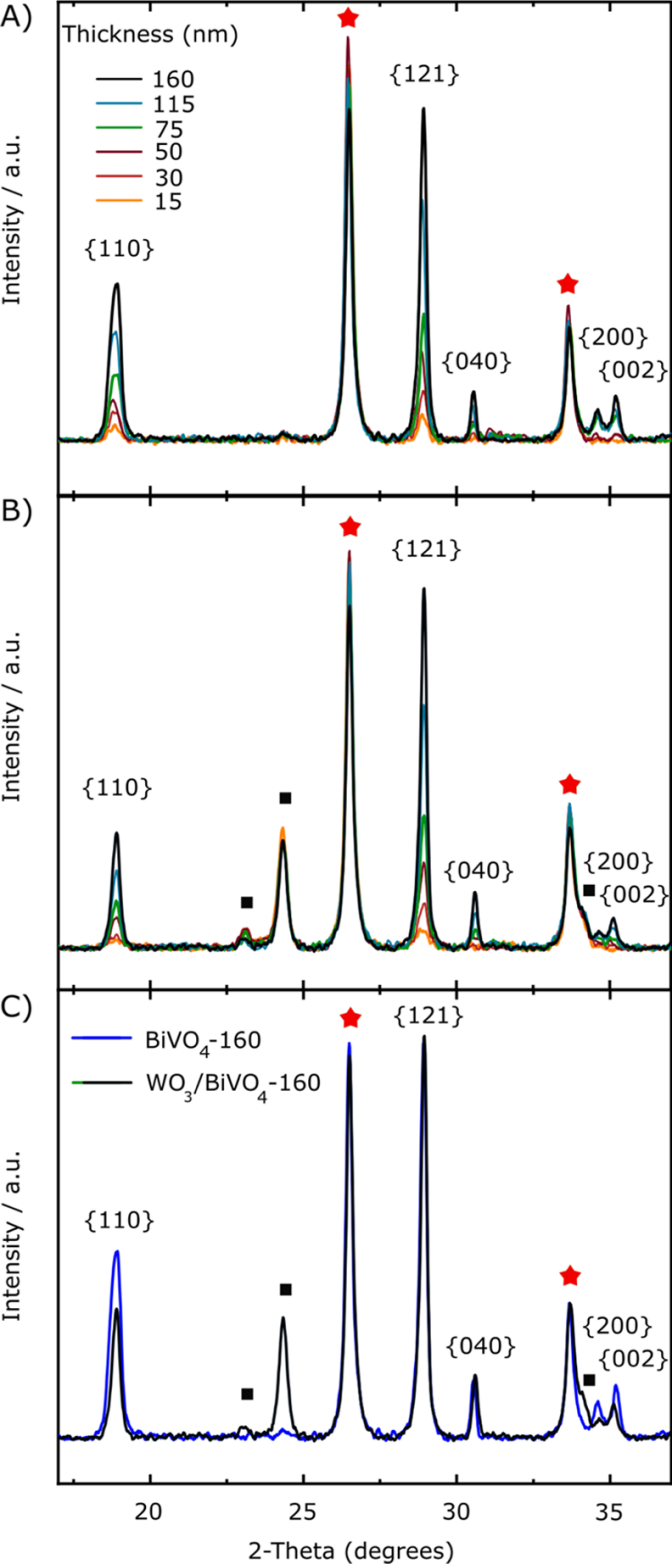
XRD analysis of (A) the BiVO_4_ and (B) the WO_3_/BiVO_4_ photoanode series with different BiVO_4_ thicknesses. (C) Comparison between the XRD patterns of BiVO_4_-160 and WO_3_/BiVO_4_-160 thickest films. Red stars and black squares mark FTO and WO_3_ patterns, respectively. The BiVO_4_ planes are indicated with their *hkl* index.

The powder XRD patterns recorded with the two photoanode series with variable BiVO_4_ thickness are shown in Figure 3. The patterns of both individual materials fit well with monoclinic (JCPDS 05-0363 for WO_3_)^48,49^ and monoclinic scheelite (JCPDS 75-1867 for BiVO_4_)^34^ structures. The XRD reflections of pure monoclinic scheelite BiVO_4_ peaking at 2θ ca. 19, 29, 31, 34.5, and 35.3° (Figure 3A) correspond to the {110}, {121}, {040}, {200}, and {002} planes, and their intensities linearly increase with increasing BiVO_4_ film thickness, as shown in Figure S2A. More specifically, the {040} reflex is associated to the BiVO_4_ {010} crystal facet, which is known to be ∼0.1 J m^–2^ more stable than the {110} one.^50,51^ These are known as the most stable monoclinic BiVO_4_ surfaces.^52^ Concerning the XRD patterns of the coupled WO_3_/BiVO_4_ systems reported in Figure 3B, the reflections assigned to monoclinic WO_3_ (black squares at 2θ = 23.0, 24.3, and 34.1°) moderately decrease with the increasing number of BiVO_4_ layers as it progressively attenuates the incident X-rays onto the underlying WO_3_. At the same time, no substantial change appears in the position of the XRD patterns belonging to scheelite BiVO_4_, with peak intensities increasing with increasing BiVO_4_ thickness.

However, when comparing the XRD patterns of the thickest BiVO_4_ films in the two series (Figure 3C), we notice that in the coupled system, the BiVO_4_ peaks at 2θ = 19, 34.5, and 35.3°, corresponding to the {110}, {200}, and {002} planes, respectively, are less intense with respect to those of the corresponding individual BiVO_4_ film. Differently, the relatively more intense peaks at 29 and 31°, associated with the most stable facets ({121} and {040} planes) of the monoclinic BiVO_4_ phase, are almost unaffected by the presence of the underlying WO_3_ coating layer and their peaks almost totally overlap in the diffraction patterns of the two thickest films in the two series (Figure 3C).

**Figure 4:**
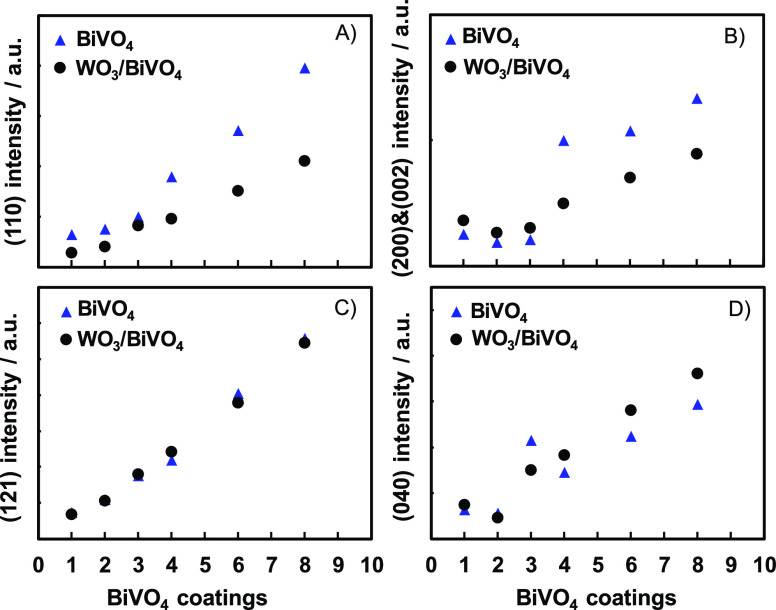
Calculated intensities of (A) {110}, (B) {200} and {002}, (C) {121}, and (D) {040} XRD reflections of BiVO_4_ in both individual (blue triangles) and coupled (black circles) series samples as a function of the number of BiVO_4_-coated layers.

This difference can be better appreciated in Figure 4, where each XRD peak intensity determined for the two investigated sample series is reported as a function of the number of BiVO_4_-coated layers. Interestingly, the main differences in the pattern intensity pertain to the {110} and {200}/{002} planes (Figure 4A,B), while the {121} and {040} planes originate similar peak intensities in the two photoelectrode series (Figure 4C,D). Thus, the presence of the WO_3_ layer appears to inhibit the BiVO_4_ crystal growth along the {110} and {200}/{002} crystallographic directions, promoting a slightly alternative structural evolution, possibly due to an additional surface stabilization occurring between WO_3_ itself and the {121} or {010} planar structures of BiVO_4_. A similar behavior has been recently observed for the interface between BiVO_4_ and graphitic carbon nitride (g-C_3_N_4_) with a preferential growth along specific directions, consequently enhancing and weakening selected XRD peaks.^53^

Aiming at better appreciating the selective WO_3_-induced alteration of the BiVO_4_ crystal growth, the ratios between the intensity of each BiVO_4_ XRD peak and that of the almost unaltered {121} pattern were calculated for all individual and coupled electrodes with the same number of BiVO_4_-coated layers. These data are collected in Table 1. Due to their extremely low intensity, such calculation could not be applied to the {200} and {002} BiVO_4_ reflections.

**Table 1:** Relative Intensity of the {110} and {040} BiVO_4_ XRD Reflections with Respect to the Almost Invariant {121} One, Calculated for Each Individual BiVO_4_ and Corresponding WO_3_/BiVO_4_ Coupled Electrode with the Same Number of BiVO_4_-Coated Layers

	{110}/{121}		{040}/{121}	
BiVO_4_ coatings	BiVO_4_	WO_3_/BiVO_4_	BiVO_4_	WO_3_/BiVO_4_
1	0.88	0.43	0.17	0.22
2	0.69	0.38	0.10	0.09
3	0.57	0.46	0.25	0.17
4	0.82	0.40	0.13	0.15
6	0.67	0.40	0.11	0.15
8	0.71	0.39	0.11	0.13

Regardless of the BiVO_4_ layer thickness, the {110}/{121} peak intensity ratios calculated for the WO_3_/BiVO_4_ electrodes are systematically ca. half those obtained for the corresponding individual BiVO_4_ films, confirming that the crystal growth along the {110} direction is partly prevented by the presence of the WO_3_ sublayer. Differently, the crystal growth of {010} planar structures appears to be unaffected by the underlying presence of WO_3_. Thus, the structure and orientation of the substrate (in the present case, WO_3_) may play a key role in controlling the phase or orientation of the subsequently deposited BiVO_4_ layers, as previously reported, e.g., in the case of monophasic TiO_2_ film deposition on SrTiO_3_ by pulsed laser ablation.^54^

### 3.2 Electronic Structure of the Interface

Aiming at getting further insights into the origin of the observed XRD peaks intensity mismatch in the two photoanode series, computational simulations were performed to shed light on (i) the nature and stability of the heterojunction at the sub-nanometer level, based on beneficial matching effects between specific crystalline facets of the two oxides, and (ii) the band edge alignment and band bending between WO_3_ and BiVO_4_.

We started from the experimental crystal structures of monoclinic WO_3_ and BiVO_4_.^55,56^ The calculated lattice vectors of WO_3_ are *a* = 7.345 Å, *b* = 7.617 Å, *c* = 7.816 Å, and β = 90.5° and compare well with the available experimental ones, *a* = 7.306 Å, *b* = 7.540 Å, *c* = 7.692 Å, and β = 90.9°.^55^ The BiVO_4_ crystal structure can be modeled employing two different settings, a body-centered *C*2/*c* cell, space group no. 15, or a *I*2/*b* one.^50,56,57^ In the first case, the experimental crystal structure is described by the lattice parameters *a* = 7.258 Å, *b* = 11.706 Å, *c* = 5.084 Å, and β = 134.1°.^50,56,57^ In the second one, the lattice parameters are *a* = 5.194 Å, *b* = 5.090 Å, *c* = 11.697 Å, and γ = 90.4°.^56,58^ In our simulations, we adopted the first scheme. The calculated (HSE06) lattice parameters *a*, *b*, *c*, and β of WO_3_ are 7.345 Å, 7.617 Å, 7.816 Å, and 90.5°, respectively. Those of BiVO_4_ are 7.215 Å, 11.544 Å, 5.093 Å, and 134.9°, respectively (deviations from the experimental values are within 1.5%, see also the Supporting Information). Then, we cut the WO_3_ and BiVO_4_ most stable surfaces starting from the relaxed bulk structures, namely, a 1.6 nm thick WO_3_ {001} slab and a 3.3 nm thick BiVO_4_ {010} slab (see Table S3 for lattice parameters).^46,50,51,59^ The convergence of the surface properties was checked against the size of the slab models.

**Figure 5:**
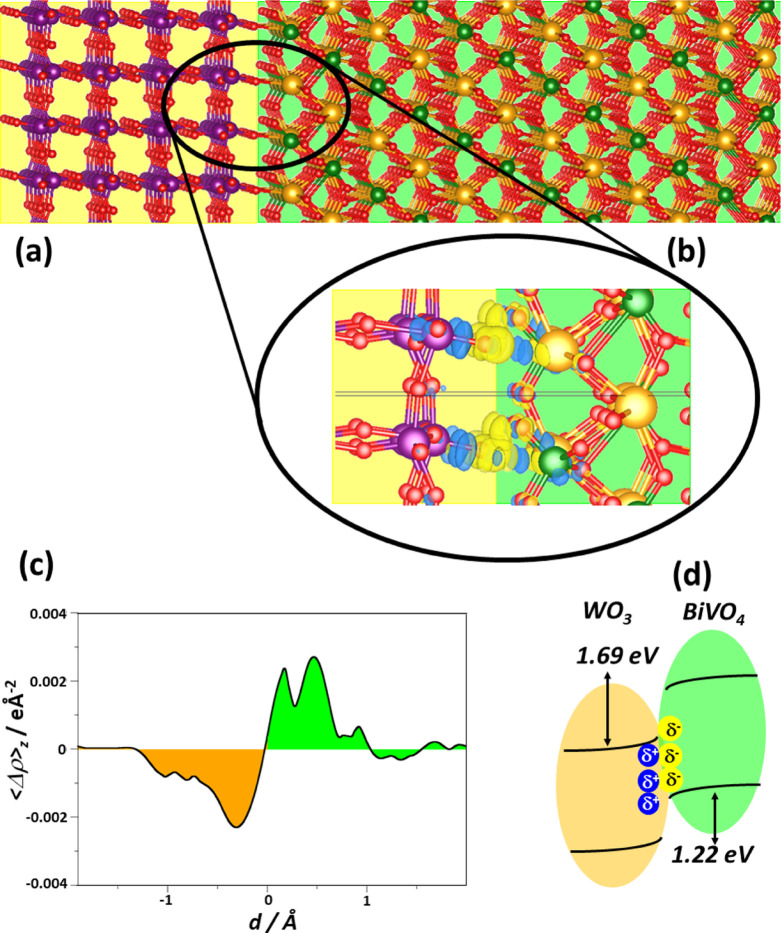
(a) Optimized WO_3_/BiVO_4_ interface model [left WO_3_ (001), right BiVO_4_ (010)]. (b) Magnified interface region which shows accumulation (yellow) and depletion (blue) regions from electron density difference (Δρ) iso-surfaces (0.01 e Å^−3^). (c) Averaged Δρ_*z*_ along planes with the same nonperiodic direction *z*. (d) Schematic view of the band alignment of the WO_3_/BiVO_4_ system according to the calculated VBOs and CBOs and interface polarization. Atoms labeling: purple: W; red: O; pink: V; and dark green: V. Orange- and light green-shaded areas surround WO_3_ and BiVO_4_ units.

A problem that arises when building an interface between two crystalline structures is that of the mismatch, which must be minimized to avoid spurious effects. We found that a 45° rotated {010} BiVO_4_ supercell displays excellent matching with the WO_3_ {001} one,^44^ including a good cation–anion matching (see Figure 5a and Table S3). This result is in line with the recently reported preferential facet orientation of WO_3_, also affecting the BiVO_4_ growth and facets at the WO_3_/BiVO_4_ heterojunction.^30^ The lattice mismatches are 1.2% and 2.1% for *a* and *b* lattice vectors, respectively, and induce a deviation of BiVO_4_ and WO_3_ electronic properties as high as 0.1 eV. Therefore, the induced mismatch can be considered low in this respect.

It must be considered that BiVO_4_ may expose the (110) surface as well, although this surface is less stable than the (010) one.^50,51^Table S3 reports the calculated surface energy which is 0.08 J m^–2^ higher than for the (010) facet. The band gap is instead very similar (difference 0.1 eV) as a consequence of the use of size-converged slab models. The same table also reports the calculated lattice vectors. The (010) surface has a nearly square lattice (*a* = 5.036 Å, *b* = 5.202 Å, and γ = 90°), while the BiVO_4_ (110) lattice is oblique, with *a* = 5.084 Å, *b* = 6.842 Å, and γ = 112.55°. This affects the possible lattice matching with WO_3_ (001). Indeed, BiVO_4_ (010) shows an excellent matching by taking a (√2), (√2) supercell, with mismatches equal to 1.1, 2.1, and 2.1% for *a*, *b*, and γ, respectively. We did several attempts to find a suitable lattice matching between BiVO_4_ (110) and WO_3_ (001), and we managed to design an interface lattice with an acceptable mismatch only by invoking very large supercells, where a 2 × 5 supercell of WO_3_ (001) matches a rotated supercell of BiVO_4_ (110) with lattice mismatches of 4.3, 2.5, and 0.8%. This cell should have lattice parameters equal to 14.620, 36.981, and 90 Å, and it is made by 2224 atoms. Unfortunately, such a large model results in too heavy computational demand at the HSE06 level. Based on stability and lattice mismatch considerations, we decided to focus on a WO_3_/BiVO_4_ interface where BiVO_4_ exposes the (010) surface.

Upon full geometry optimization, new chemical bonds form at the interface, as evidenced in Figure 5b. In particular, Bi–O bonds (*d* = 2.73 Å) are preferentially formed, rather than V–O ones (*d* = 3.29 Å), while the W–O bond distance in the contact region (1.69 Å) is the same as in WO_3_ (1.70 Å).

The energetics of the interface was evaluated by calculating the interfacial energy (*E*_form_), that is, the formation energy of the interface from the bulk materials.^60^*E*_form_ = 0.66 J m^–2^ indicates a high stability of the interface.^61^ Furthermore, the formation of the interface from the separated WO_3_ and BiVO_4_ slabs implies an energy gain by 0.13 J m^–2^, as evidenced by the calculated adhesion energy *E*_ad_, defined as the energy of the interface minus that of the separated slabs.

Concerning the electronic properties, interface polarization and the band alignment of the WO_3_/BiVO_4_ interface, the calculated band gap of WO_3_ is 2.98 eV, close to the experimental estimates (2.6–3.2 eV);^55,62^ the band gap of BiVO_4_ (3.23 eV) is overestimated by about 0.7 eV with respect to the experimental value but in line with calculated values with the same functional.^38,63^ The band gaps of WO_3_ (001) and BiVO_4_ (010) slabs are 2.86 and 3.33 eV, thus almost converged to the bulks’ values (see Table S3 for full details). We evaluated the interface polarization (Δ*q*) and the direction of the band bending, an important aspect to determine the preferred location for photogenerated electrons and holes.^64,65^ We estimated Δ*q* in two different ways using the Mulliken charges and the charge density difference Δρ, defined as Δρ = ρ_WO_3_/BiVO_4__ – ρ_WO_3__ – ρ_BiVO_4__, and the integrated plane-averaged charge density difference ⟨Δρ⟩_*z*_. The two approaches give the same qualitative trend with Δ*q* = 0.2 e nm^–2^ by using Mulliken charges and 0.1 e nm^–2^ by using ⟨Δρ⟩_*z*_. The analysis shows that an electron polarization occurs from WO_3_ to BiVO_4_ due to the interface formation (see also Figure 5c), which shows a charge depletion on WO_3_ and a charge accumulation on BiVO_4_.

The VB and CB offsets (VBOs, CBOs) (Figure 5d) were calculated following the potential line-up procedure used in previous works^47,66,67^ and briefly described in the Supporting Information. The system is characterized by a type-II heterojunction, thus favorable for charge-carrier separation,^68−70^ in line with Mott–Schottky analyses.^71^

The predicted type-II alignment is in line with the measured band edges of WO_3_ and BiVO_4_ materials^17^ (Table S5). The interface formation is expected to be beneficial for carrier separation because both (i) band edge alignment and (ii) interface polarization suggest that photopromoted electrons should migrate from BiVO_4_ to WO_3_ and photogenerated holes in the opposite direction, thus providing a first-principles rationalization of the experimental observations.

The formation of the interface leads to a ∼0.3 eV enhancement of the band offsets, which is expected to improve the driving force of the electron migration toward WO_3_ and holes migration toward BiVO_4_. This 0.3 eV band offset increase is similar to that obtained in oxide interfaces.^72^

The convergence of the results as a function of the BiVO_4_ layer thickness was investigated in order to exclude quantum confinement effects.^73^ We simulated two additional interface models (full data in the Supporting Information) having different BiVO_4_ thicknesses, while the thickness of WO_3_ was kept fixed to 1.6 nm since it already leads to converged properties. We reduced the BiVO_4_ thickness from 3.3 to 2.2 and 1.1 nm, respectively (see Table S4). Lattice constants and adhesion energies are very close in the three models. Also, the band alignment remains unchanged and the VBO and CBO deviate by less than 0.1 eV. Therefore, from a purely thermodynamic point of view, the electronic properties of WO_3_/BiVO_4_ are converged with a BiVO_4_ film thickness of 2–3 nm, which is far below the thickness of the thinnest BiVO_4_ layer (15 nm) in our photoanodes.

### 3.3 PEC Characterization

**Figure 6:**
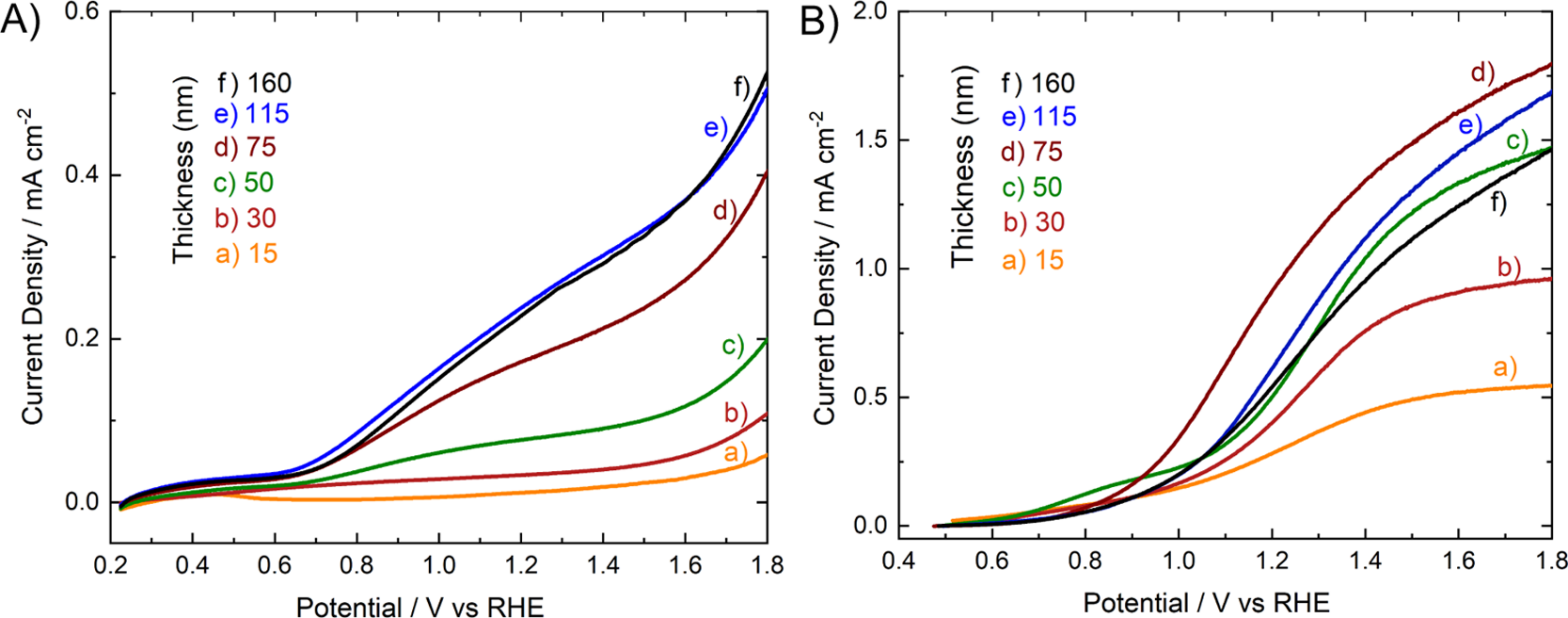
LSV curves under AM 1.5 G irradiation of (A) the BiVO_4_ and (B) the WO_3_/BiVO_4_ photoanodes recorded under backside irradiation in 0.5 M Na_2_SO_4_ aqueous solutions.

The LSV curves recorded under backside irradiation (irradiation on the FTO side) with individual BiVO_4_ or with the corresponding coupled WO_3_/BiVO_4_ photoanodes with different BiVO_4_ thicknesses can be compared in Figure 6 in terms of photocurrent density versus applied potential (*J*–*V*) plots.

The LSV response obtained from the heterojunction photoanodes was much higher than that recorded with the corresponding single BiVO_4_ photoanodes with the same thickness of the BiVO_4_ layer. The two types of photoanodes exhibit a photocurrent increasing with increasing BiVO_4_ thickness for BiVO_4_ thicknesses in the 15–75 nm range but a different behavior for a relatively higher BiVO_4_ layer thickness. In fact, while in single BiVO_4_ photoanodes, the photocurrent density increases with the BiVO_4_ thickness up to 115 nm, and almost the same LSV curve is obtained with BiVO_4_-115 and BiVO_4_-160 (Figure 6A), in the case of the WO_3_/BiVO_4_ coupled system the highest photocurrent density is attained with WO_3_/BiVO_4_-75, having a 75 nm thick BiVO_4_ layer, while progressively lower LSV curves are recorded with the thicker WO_3_/BiVO_4_-115 and the WO_3_/BiVO_4_-160 photoanodes (Figure 6B).

**Figure 7:**
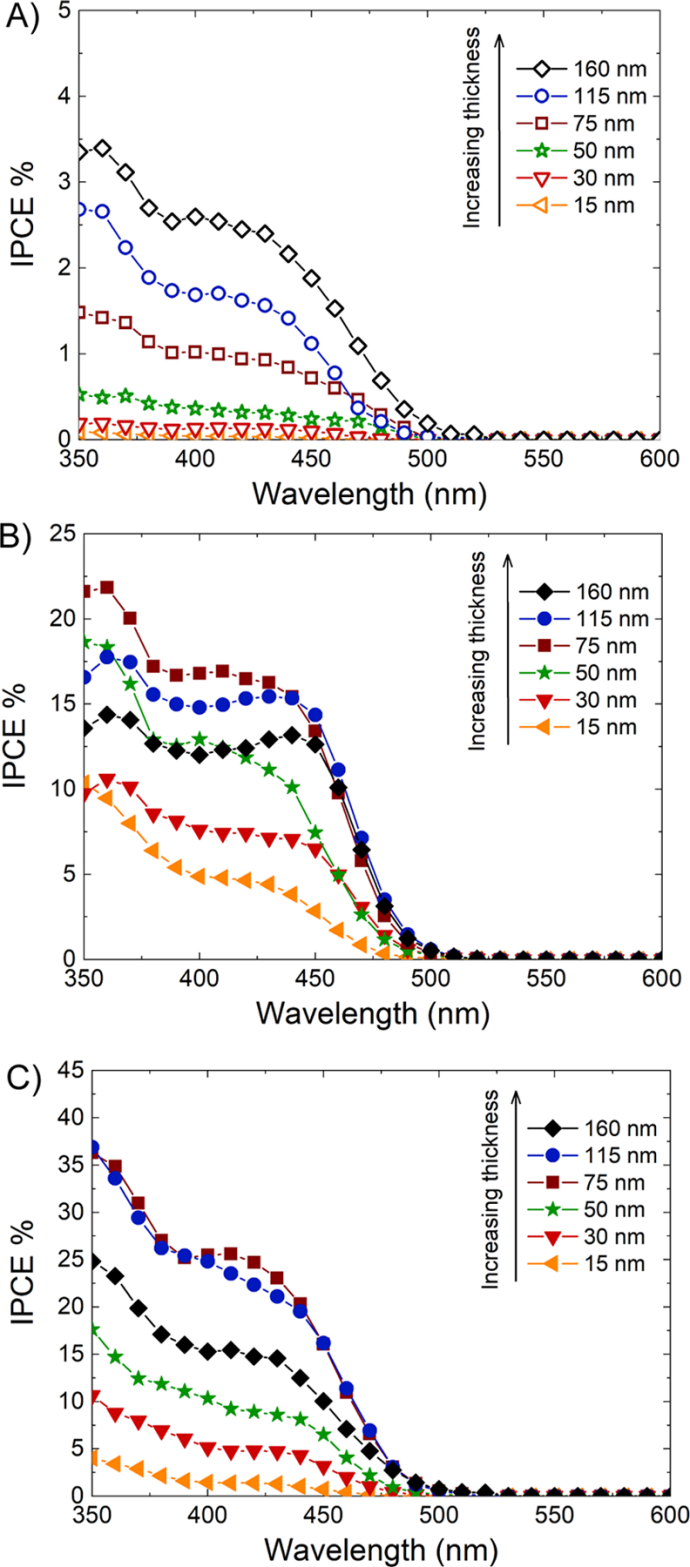
IPCE curves of (A) the BiVO_4_ and (B) the WO_3_/BiVO_4_ photoanode series under backside irradiation and of (C) the WO_3_/BiVO_4_ photoanode series under frontside irradiation, recorded in Na_2_SO_4_ 0.5 M aqueous solution under a 1.23 V vs RHE applied bias.

The PEC properties of the two series of electrodes were also investigated by IPCE tests at 1.23 V versus RHE in a Na_2_SO_4_ 0.5 M electrolyte solution. The IPCE curves obtained under backside irradiation with all photoanodes, reported in Figure 7A,B, show that, in general, BiVO_4_ is active up to 520 nm and that in pure BiVO_4_ photoanodes (Figure 7A) the IPCE increases with the increasing thickness of the BiVO_4_ layer, in line with the results of LSV analysis shown in Figure 6A.

On the other hand, all heterojunction electrodes (Figure 7B) outperform the IPCE of the thickest BiVO_4_ film. Indeed, the charge separation occurring in the WO_3_/BiVO_4_ electrodes and the superior charge-transport properties of WO_3_ with respect to BiVO_4_ allow us to achieve a higher IPCE by limiting the charge-carrier recombination.^28,74,75^ In this case, the IPCE increases with increasing BiVO_4_ film thickness only for the thinner BiVO_4_ layers in the composite system, the WO_3_/BiVO_4_-75 electrode exhibiting the highest efficiency (Figure 7B), while thicker WO_3_/BiVO_4_-115 and WO_3_/BiVO_4_-160 composite photoanodes become progressively less efficient at wavelengths below 450 nm. Also, these results are fully compatible with the results of LSV analyses shown in Figure 6B.

When photoanodes are irradiated from the FTO side, most of the charge carriers are photogenerated in BiVO_4_ close to the WO_3_ layer. In particular, the so-produced holes need to be transferred through the BiVO_4_ layer to reach water molecules at the electrode/electrolyte interface. Along this way, they can recombine with photopromoted electrons trapped in BiVO_4_, and the probability that they reach the electrode/electrolyte interface decreases with the increasing thickness of the BiVO_4_ layer.

The wavelength dependence of the IPCE curves recorded with WO_3_/BiVO_4_ electrodes is consequent to the fact that the WO_3_ layer absorbs light below 450 nm (see Figure 2B). Under such conditions, the holes photogenerated in BiVO_4_ close to the WO_3_/BiVO_4_ heterojunction may partly recombine with electrons photopromoted in the CB of WO_3_, as demonstrated by ultrafast transient absorption measurements in our previous study.^63^

These effects become more evident with the increasing thickness of the BiVO_4_ layer in WO_3_/BiVO_4_ photoanodes, producing a change in the shape of the IPCE curves (Figure 7B) with the relative photon-to-current efficiency at a shorter wavelength monotonously decreasing with increasing BiVO_4_ layer thickness. Indeed, in the case of the WO_3_/BiVO_4_-15 photoanode, having the thinnest (15 nm thick) BiVO_4_ layer so that holes are also photoproduced close to the electrode/electrolyte interface, the IPCE is by far maximum at the shortest wavelengths, though its average value is lower than in thicker photoanodes due to the lower amount of absorbed light.

The recombination between CB electrons in WO_3_ and VB holes in BiVO_4_ at the WO_3_/BiVO_4_ heterojunction becomes much less important if the electrodes are irradiated from the front side (through the BiVO_4_/electrolyte interface). In fact, the IPCE curves recorded with the WO_3_/BiVO_4_ electrodes under frontside irradiation (Figure 7C) are almost doubled with respect to those recorded under backside irradiation (Figure 7B), with relatively higher IPCE values especially at wavelengths shorter than 450 nm and for coupled systems containing a BiVO_4_ layer thicker than 50 nm, that is, under conditions of relatively low excitation of the WO_3_ underlayer.

The heterojunction film with a BiVO_4_ thickness of 75 nm presents (under both irradiation configurations) the optimal balance between visible light absorption and low charge-carrier recombination during hole transfer to the electrode/electrolyte interface, and this leads to maximum IPCE.

Finally, the much better performance of composite WO_3_/BiVO_4_-15 with respect to single BiVO_4_-15 (Figure 6) confirms what predicted by DFT calculations, that is, the stabilization of a type-II heterojunction is able to promote an effective electron–hole separation in coupled WO_3_/BiVO_4_ systems with an at least 3 nm thick BiVO_4_ layer.

## 4 Conclusions

In conclusion, we tuned the visible light absorption of the WO_3_/BiVO_4_ heterojunction by increasing the BiVO_4_ thickness and observed that the WO_3_ underlayer impacts the BiVO_4_ crystal structure, in particular on the {010} plane. DFT calculations support that the two oxides are joint at the WO_3_ {001} and BiVO_4_ {010} interface and that the heterojunction leads to a type-II band alignment and a charge polarization, which are beneficial for charge separation. The observed wavelength-dependent PEC performance of the composite WO_3_/BiVO_4_ photoanodes with differently thick BiVO_4_-coated layers results from the relatively poor charge-transport properties of the BiVO_4_ layers and the probability that holes photoproduced in BiVO_4_ close to the interface between the two oxides recombine with electrons photopromoted in the WO_3_ CB upon irradiation at wavelengths below 450 nm. The WO_3_/BiVO_4_ electrode with a 75 nm thick BiVO_4_ layer is best performing, with an optimal balance between thickness-dependent effective light absorption and charge-carrier recombination. These results deepen the insights into this heterojunction and may suggest a strategy to develop efficient visible-light-harvesting systems.

## Supporting Information Available

Cross-sectional FESEM images and extinction coefficient of BiVO_4_, supporting XRD analyses, and supporting DFT calculations (PDF)
