# Supplementary material for: WO3/BiVO4 Photoanodes: Facets Matching at the Heterojunction and BiVO4 Layer Thickness Effects
Source: ACS Appl Energy Mater. 2021 Aug 12;4(8):8421–31. doi: 10.1021/acsaem.1c01623 (PMC8414527; doi:10.1021/acsaem.1c01623)
Supplement: Supplementary file 1 — ae1c01623_si_001.pdf [file ae1c01623_si_001.pdf]

SUPPORTING INFORMATION

WO_3/$BiVO_4$ Photoanodes: Facets Matching at the Heterojunction and $BiVO_4$ Layer Thickness Effects

Ivan Grigioni,[†,ǀ] Giovanni Di Liberto, [‡,ǀ] Maria Vittoria Dozzi,[†] Sergio Tosoni,[‡] Gianfranco Pacchioni,[‡] and Elena Selli*,[†]

[†] Dipartimento di Chimica, Università degli Studi di Milano, Via Golgi 19, 20133 Milano, Italy

[‡] Dipartimento di Scienza dei Materiali, Università di Milano-Bicocca, via Cozzi 55, 20125 Milano, Italy

[ǀ] These authors contributed equally

* Corresponding author
E-mail: elena.selli@unimi.it

1. Cross sectional FESEM images and extinction coefficient of $BiVO_4$
2. Supporting XRD analyses
3. Supporting DFT calculations

1. Cross section FESEM images and extinction coefficient of $BiVO_4$

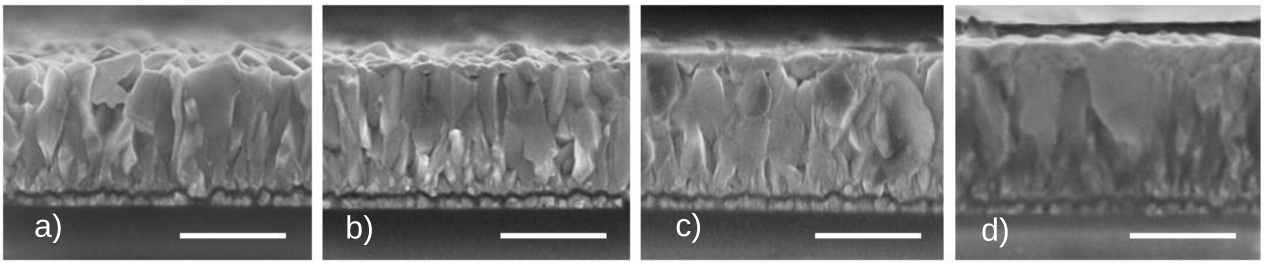

Figure S1. Cross section FESEM images (a) of clean FTO and of $BiVO_4$ photoanodes prepared by successive deposition of (b) 2, (c) 4 and (d) 6 $BiVO_4$ layers. The scale bar is 500 nm.

We performed the FESEM cross section analysis of the films prepared by 2, 4 and 6 successive $BiVO_4$ depositions (Figure S1); the cross section of the clean conductive glass substrate was used to determine the thickness of the pristine FTO layer. From the absorption spectra of the three electrodes, reported in Figure 2, and the evaluated thickness of the $BiVO_4$ overall layers in $BiVO_4$ photoanodes, we calculated the absorption coefficient of $BiVO_4$ at 420 nm (α_{420}) using the following equation: $A_{420} = \alpha_{420} \times \mathrm{d}$, where d is the average thickness in cm and A_{420} the absorbance at 420 nm (Figure 2A). The absorption coefficient α_{420} = 6.7 10^4 cm^{-1} was obtained as the slope of the absorbance at 420 nm *vs.* film thickness plot shown below.

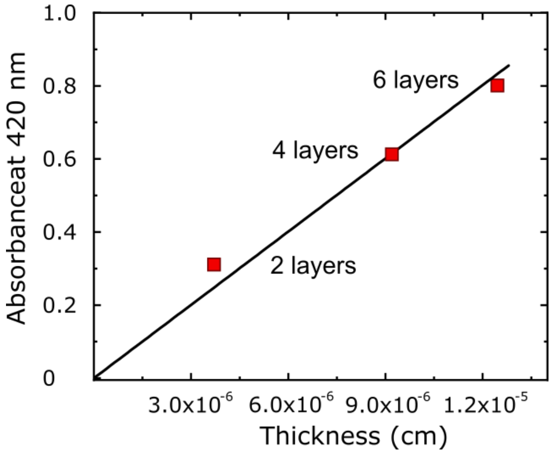

The calculated α_{420} allowed us to estimate the thickness of the individual $BiVO_4$ films (either deposited on FTO or on FTO/WO_3) from the electrode absorbance at 420 nm. The thickness of the $BiVO_4$ layer was found to increase almost linearly with the number of coated layers with an average increment of *ca.* 20 nm per coated layer, as shown by the data reported in Table S1.

Table S1. Absorbance (Abs) at 420 nm and thickness of the $BiVO_4$ layers in single $BiVO_4$ and composite WO_3/$BiVO_4$ photoanodes, obtained upon deposition of a different number of $BiVO_4$ coated layers.

	$BiVO_4$		WO_3/$BiVO_4$	
Coatings	Abs	Thickness (nm)	Abs	Thickness (nm)
1	0.09	14	0.11	16
2	0.21	31	0.20	30
3	0.33	49	0.33	50
4	0.50	75	0.50	74
6	0.76	114	0.77	115
8	1.09	163	1.07	160

2. Supporting XRD analyses

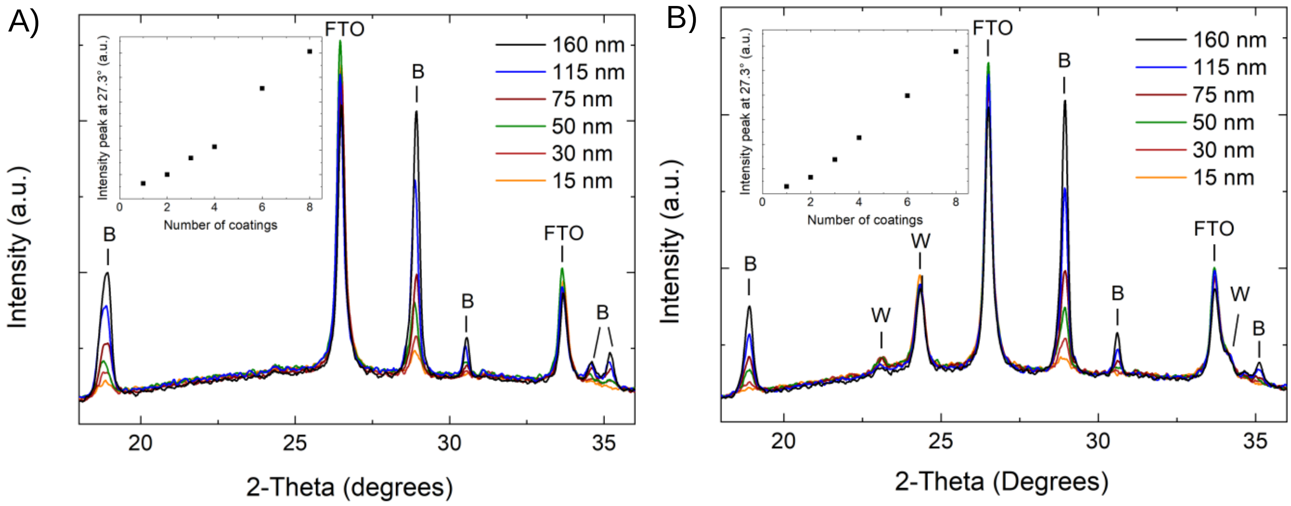

Figure S2. XRD patterns of (A) the $BiVO_4$ and (B) the WO_3/$BiVO_4$ photoanodes series. The reflections of $BiVO_4$ and WO_3 monoclinic scheelite, as well as those of FTO, are indicated with B, W and FTO, respectively. Insets: intensity of the peak at 27.3° vs. the number of coated $BiVO_4$ layers.

3. Supporting DFT calculations

3.1 Cutoff limits, convergence criteria and thresholds, and reciprocal space sampling

The cutoff limits in the evaluation of Coulomb and exchange series were set to 10^{-7} for both Coulomb overlap and penetration, 10^{-7} for exchange overlap and pseudo-overlap in the direct space, and 10^{-14} for exchange pseudo-overlap in the reciprocal space. An SCF was considered converged when the energy difference between two consecutive steps was below 10^{-6} au. The reciprocal space of heterostructure models was sampled by adopting a working shrinking factor equal to 4. For all atoms, the thresholds for the maximum and the root mean square forces were set to 0.00045 and 0.00030 au and those for the maximum and the root mean square atomic displacements to 0.00180 and 0.00120 au, respectively.

3.2 Results

a. Bulk WO_3 and $BiVO_4$

The calculated bulk structural and electronic properties of WO_3 and $BiVO_4$ are reported in Table S2, in comparison with available experimental data.

Table S2. Optimized lattice parameters (*a*, *b, c, β*), and band gap (E_g) of WO_3 and $BiVO_4$.

	a / Å	*b* / Å	*c* / Å	*β* / degrees	E_g / eV
WO_3 [DFT]	7.345	7.617	7.816	90.5	2.98
WO_3 [Exp.]	7.306	7.540	7.692	90.9	2.6-3.2
$BiVO_4$ [DFT]	7.215	11.544	5.093	134.9	3.23
$BiVO_4$ [Exp.]	7.258	11.706	5.084	134.1	2.4-2.5

b. WO_3 (001) and $BiVO_4$ (010) surfaces

The fully optimized bulk structures have been used to design surface models. We designed a WO_3 (001) 1.6 nm thick slab which ensures a good convergence of surface properties,

and variously sized $BiVO_4$ (010) slabs, with a thickness ranging from 1.1 to 3.3 nm. The calculated structural and electronic properties of fully optimized slabs models are reported in Table S3.

Table S3. Relaxed lattice parameters (a, b, γ), surface energy (E_s , with respect to the bulk) and band gap (E_g) of WO_3 and $BiVO_4$ slabs used to design the interface models.

system	Thickness / nm	a / Å	b / Å	γ / degrees	E_s / J m^{-2}	E_g / eV
WO_3 (001)	1.6	7.311	7.382	90.0	0.37	2.86
$BiVO_4$ (010)	1.1	4.989	5.258	89.5	0.35	3.51
	2.2	5.023	5.219	89.8	0.36	3.38
	3.3	5.036	5.202	90.0	0.37	3.33
	3.3 (√2; √2)[a]	7.240	7.240	88.1	0.37	3.33
$BiVO_4$ (110)	2.8	5.084	6.842	112.6	0.45	3.23

[a] This cell is obtained by a rotation of 45° with respect to the standard crystallographic orientation.

c. WO_3/$BiVO_4$ interfaces models

The WO_3 (001) slabs have been interfaced with the three $BiVO_4$ (010) models described above. All composite models have been fully optimized. The calculated lattice parameters are reported in Table S4, together with the calculated adhesion energy E_{ad}, defined as:

$$E_{ad} = E_{WO3/BiVO4} - (E_{WO3(001)} + E_{BiVO4(010)})$$

Table S4. Optimized lattice parameters (a, b, γ), surface area (A) and adhesion energy (E_{ad}) of the heterojunction models with increasing $BiVO_4$ thickness.

WO_3/$BiVO_4$ thickness / nm	a / Å	b / Å	γ / degrees	A / Å^2	E_{ad} / J m^{-2}
2.7	7.276	7.292	92.0	53.0	-0.12
3.8	7.267	7.267	91.8	52.8	-0.13
5.0	7.262	7.255	91.7	52.7	-0.13

d. Band Alignment of WO_3/$BiVO_4$ interfaces models

The valence band (VBM_{WO3}, VBM_{BiVO4}) and conduction band (CBM_{WO3}, CBM_{BiVO4}) edges of independent components can be aligned by using, as a common reference, the calculated plane averaged electrostatic potential (V) along the non-periodic dimension z.[1–4] In particular, the stationary points of V (V^{TOP}) in the interplanar regions of each isolated unit (V^{TOP}_{WO3}, V^{TOP}_{BiVO4}) are compared to those of the interface ($V^{TOP}_{WO3,Het}$, $V^{TOP}_{BiVO4,Het}$). In this way, the band structures are aligned to a common reference energy, permitting to evaluate the shift induced by the interfacial effects. The valence band offset (VBO) and conduction band offset (CBO) can hence be calculated as:

$$VBO = (VBM_{WO3} - V^{TOP}_{WO3}) - (VBM_{BiVO4} - V^{TOP}_{BiVO4}) + (V^{TOP}_{WO3,Het} - V^{TOP}_{BiVO4,Het})$$

$$CBO = (CBM_{WO3} - V^{TOP}_{WO3}) - (CBM_{BiVO4} - V^{TOP}_{BiVO4}) + (V^{TOP}_{WO3,Het} - V^{TOP}_{BiVO4,Het})$$

Table S5. Calculated *VBO* and *CBO* of WO_3/$BiVO_4$ interfaces based of separated units and explicit interface calculations. The minus sign indicates that WO_3 band edges are lower in energy that the same of $BiVO_4$.

WO_3/$BiVO_4$ thickness / nm	separated		interface	
	VBO / eV	*CBO* / eV	*VBO* / eV	*CBO* / eV
5.0	-0.9	-1.31	-1.22	-1.69

The formation of the interface leads to a ~0.3 eV enhancement of the band offsets, which is expected to improve the driving force of the electrons migration toward WO_3 and holes migration towards $BiVO_4$. This 0.3 eV band offsets increase is similar to what obtained in oxide interfaces.[5]

References

(1) Cerrato, E.; Gionco, C.; Paganini, M. C.; Giamello, E.; Albanese, E.; Pacchioni, G.

Origin of Visible Light Photoactivity of the CeO_2/ZnO Heterojunction. *ACS Appl. Energy Mater.* **2018**, *1*, 4247–4260. https://doi.org/10.1021/acsaem.8b00887.

(2) Van de Walle, C. G.; Martin, R. M. Theoretical Study of Band Offsets at Semiconductor Interfaces. *Phys. Rev. B* **1987**, *35*, 8154–8165. https://doi.org/10.1103/PhysRevB.35.8154.

(3) D'Amico, N. R.; Cantele, G.; Ninno, D. First Principles Calculations of the Band Offset at $SrTiO_3-TiO_2$ Interfaces. *Appl. Phys. Lett.* **2012**, *101*, 141606. https://doi.org/10.1063/1.4757281.

(4) Conesa, J. C. Modeling with Hybrid Density Functional Theory the Electronic Band Alignment at the Zinc Oxide-Anatase Interface. *J. Phys. Chem. C* **2012**, *116*, 18884–18890. https://doi.org/10.1021/jp306160c.

(5) Conesa, J. C. Computing with DFT Band Offsets at Semiconductor Interfaces: A Comparison of Two Methods. *Nanomaterials* **2021**, *11*, 1581. https://doi.org/10.3390/nano11061581.
